# Are there over 200 distinct types of interstitial lung diseases?

**DOI:** 10.1186/s12931-024-02734-0

**Published:** 2024-03-25

**Authors:** Joseph C. Cooley, Evans R. Fernández Pérez

**Affiliations:** Division of Pulmonary, Critical Care and Sleep Medicine, National Jewish Health, Denver, CO USA

Interstitial lung diseases (ILD) are a heterogeneous group of complex disorders with varying presentations, prognoses, and responsiveness to drugs. The review by Amati and colleagues elegantly illustrates how historically ILD recognition has evolved from a handful group of histological categories described in the 1960s to the 1990s first radiological-histological classification to today’s collection of distinct clinical diagnoses. However, the study by Amati and colleagues, [1] along with numerous other articles, cite that there are over 200 ILDs. This staggering statistic has become a common introductory catchphrase for pharmaceutical and ILD educational websites. Moreover, if in doubt, check your medical center’s website. The exact verbiage varies from subtypes, disorders, disease entities, conditions, types, forms, or varieties of ILD. The number is proliferating like mushrooms in the middle of the night and has increased over time, from 100 [2] to 150 [3, 4] to over 200 [5, 6] unique ILDs.

In search of the primary data to support this claim, we followed the trail of breadcrumbs through citations. Each cited paper would make the claim of over 200 ILDs in their abstract or introduction, then reference another article that did the same, and so on. Thus, where did it start? Our search through published literature led us as far back as a case report from 1988 claiming that there are over 100 ILDs [7]. But alas, no primary data. We reached out to the eldest, wisest, and grayest ILD physicians we know, but still no clear primary data source.

Similar clinical, radiological, pathological and corresponding molecular patterns can occur in a variety of ILDs and different ones can develop within an isolated ILD. However, during the diagnostic process, the final clinical diagnosis is dictated by the integration of each of these sometimes discordant domains in the context of multidisciplinary consensus—therefore, the finite number of ILD clinical diagnoses. Using standard classification schemes of ILD clinical diagnoses, the number of unique ILDs is far less than 200. However, the number grows exponentially as we add overlapping clinical, imaging or histopathological features (e.g., interstitial pneumonia with features of autoimmunity or coexisting histological fibrotic nonspecific interstitial pneumonia and usual interstitial pneumonia) or phenotypic descriptors allowing important prognostic (e.g., attributable risk factors associated with progressive pulmonary fibrosis) and treatment distinctions as diagnostic subgroups with distinct clinical outcomes—case in point: rheumatoid arthritis-related interstitial lung disease (RA-ILD)—e.g., usual interstitial pneumonia (RA-UIP), nonspecific interstitial pneumonia (RA-NSIP), or organizing pneumonia (RA-OP). In the case of exposure-related ILD such as hypersensitivity pneumonitis, however, the different clinical descriptive terms (e.g., farmer’s lung, bird fancier’s lung) referring to numerous antigenic types and sources should not be confused with each one always causing a singular ILD clinical diagnosis. The same applies to terms used to describe primary airway-centered disorders, sparing the interstitium, listed as individual ILDs by many book chapters and reviews, demonstrating the same histological findings resulting from various of insults.

Fortunately, the number of individual types of ILDs commonly encountered by ILD specialists during their career does not exceed 200 by a long chalk. As the complexities of ILDs and their impact on patient’s lives are better understood, it is also encouraging to know that the implementation of a comprehensive pharmacological and non-pharmacological ILD bundle will continue to evolve and become increasingly patient-centric and refined over time.

## Data Availability

Data sharing is not applicable to this article as no datasets were generated or analyzed during the current study.
